# High Conversion of Styrene, Ethylene, and Hydrogen to Linear Monoalkylbenzenes

**DOI:** 10.3390/molecules23061260

**Published:** 2018-05-25

**Authors:** David Hermann Lamparelli, Antonio Ricca, Vincenzo Palma, Leone Oliva

**Affiliations:** 1 Dipartimento di Chimica e Biologia “Adolfo Zambelli”, Università di Salerno, I-84084 Fisciano, Italy; dlamparelli@unisa.it; 2 Dipartimento di Ingegneria Industriale, Università di Salerno, I-84084 Fisciano, Italy; aricca@unisa.it (A.R.); vpalma@unisa.it (V.P.)

**Keywords:** poly-insertion catalysis, ansa-metallocene, LAB, styrene regiochemistry

## Abstract

1-Alkylbenzenes as a precursor of surfactants, can be produced from ethylene, styrene, and hydrogen. These intermediates, lacking tertiary carbons, are environmentally more benign than commercial ones that bear the aromatic ring linked to an internal carbon of the aliphatic chain. The one-pot synthesis of highly linear 1-alkylbenzenes (LABs) through the homogeneous catalysis of olefin poly-insertion from cheap and largely available reagents can be carried out with a high turnover and selectivity. A purposely designed reactor that allows for the fine control of the three components feed, along with temperature, plays a key role in this achievement. A turnover of 194 g of LABs per mmol of catalyst per hour can be obtained with the simultaneous removal of polyethylene as a by-product.

## 1. Introduction

Anionic surfactants, with a worldwide production of some millions of tonnes are produced through the sulfonation of alkylbenzenes, are of relevant commercial interest. In particular, Linear AlkylBenzenes (LAB) are preferred to the branched ones, as they are eco-friendly products due to their faster and more complete biodegradation. Currently, these intermediates are industrially produced through the Friedel−Crafts alkylation of benzene with normal C10–C16 chloroalkanes or olefins [1,2,3,4], affording LABs with the aromatic ring, and occupying different positions in the aliphatic chain [2,3]. An alternative route using biomass derivatives still seems far from any practical application [5].

A recent paper [6] proposed the homogeneous Ziegler-Natta catalysis as a tool for the direct synthesis of LABs from commodities, through a dramatically simplified process when compared to those generally used. In light of the knowledge of styrene-ethylene reactivity [7], a synthesis that takes together styrene, ethylene, and hydrogen has been reported, resulting in a mixture of linear 1-alkylbenzenes with an average length of the aliphatic tail of around 14 carbon atoms. This was the result of a careful selection among the potential coordination frameworks of the catalytic metal, in order to avoid the formation of branches that are detrimental for the biodegradability of the detergents and minimize the yield of by-products such as alpha-omega DiPhenyl Alkanes (α–ω DPA), with the yields reported and overall process seeming unsuitable for a practical application. Efforts to transform these preliminary results into a real process with a potential practical application, reported in this work, have been made using a purposely designed reactor and by fine tuning the gaseous feed. 

## 2. Results

The metal–organic compound used as a catalyst is Ethylenebis(Tetrahydroindenyl) Zirconium Dichloride (EBTHIZrCl_2_) activated by Modified MethylAluminOxane (MMAO), a formulation very close to the commercial catalysts used for polymerization processes. Studies [6] have assigned EBTHIZrCl_2_ as the catalytic complex that more effectively balances activity and selectivity. 

For comparison purposes in Exp. 1, the previously described approach was reproduced by using an extremely simple commercial reactor, made up of a steel tube surmounted with a pressure gauge, with a pin tap and an aluminium safety membrane with 10 MPa breaking; the reaction was carried out under isothermal conditions, through a thermostated warming wrap. A magnetic stirring assured a uniform species concentration in the liquid phase. With respect to the experimental procedure described elsewhere [5], the batch pressure was reduced to 1 MPa, and the gas phase was composed by an ethylene and hydrogen mixture, with a H_2_/C_2_H_4_ molar ratio equal to 1. Under these conditions (T = 70 °C, p_C2H4_ = 0.5 MPa), it is possible to estimate the concentration of ethylene in the solution as being around 0.37 M, according to the data in current literature [8]. In order to compensate the pressure reduction due to the gaseous reactants conversion, a C_2_H_4_ line, fixed to 1 MPa, was connected to the reactor. On the one hand, such a solution assured a constant operating pressure inside the reaction cylinder; on the other hand, the conversion of the hydrogen and ethylene was compensated by only ethylene. Therefore, throughout the tests, the gas phase composition was not constant, since the hydrogen volumetric fraction decreased. In order to keep the composition of the gas phase constant, the reaction was stopped after 1 h, at a low feed conversion. Among the reaction products in addition to the linear alkyl-benzenes and α–ω DPA, a polymer material was recovered as a by-product whose melting temperature and the X-ray powder diffraction spectrum suggest a linear structure of polyethylene (HDPE, see supplementary materials).

**Figure 1:**
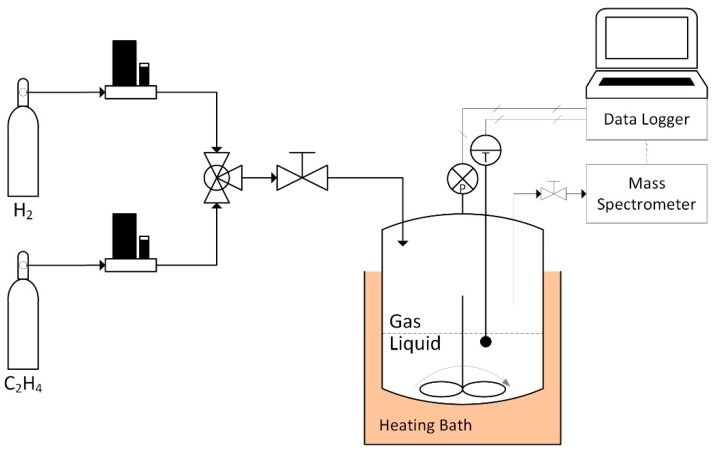
Scheme of the batch reactor and the related control scheme.

Further experiments (Exp. 2, Exp. 3) were carried out in a novel reaction system, designed with the aim of maximizing the mass transfer between the liquid and gaseous phases, and equipped to have an overall control of the gaseous phase (Figure 1). The apparatus was designed to continuously and independently feed hydrogen and ethylene, in order to compensate the pressure decrease owing to the gaseous reactants conversion, as well as to ensure a constant gas composition in the reactor. The total pressure was held at 1 MPa, and the composition of the gaseous mixture was fixed at 52% H_2_, 48% C_2_H_4_. The system temperature control was achieved through a thermostatic bath with high temperature oil. In the cylindrical batch reactor, the zirconocene complex was dissolved in 200 mL of a mixture 7/13 *v*/*v* of styrene and toluene containing the MMAO with an [Al]/[Zr] ratio of about 400.

In Exp. 2, the reaction system was set to the desired operating conditions, after which the reaction was trigged by putting the catalyst (11.0 mg) into the reaction vessel. After 2.5 h, the gaseous mixture was vented off, and the liquid phase was chemically stabilized with ethanol (200 mL) acidified with 4 mL of 37% hydrochloric acid.

The instrumental control carried out during the experiment showed that, under the conditions adopted, the reaction rate is very high. Consequently, the exothermicity of the process led to a relevant raising of the temperature that in turn induces a loss of the kinetics control, thus affecting both selectivity and catalyst lifetime. 

**Figure 2:**
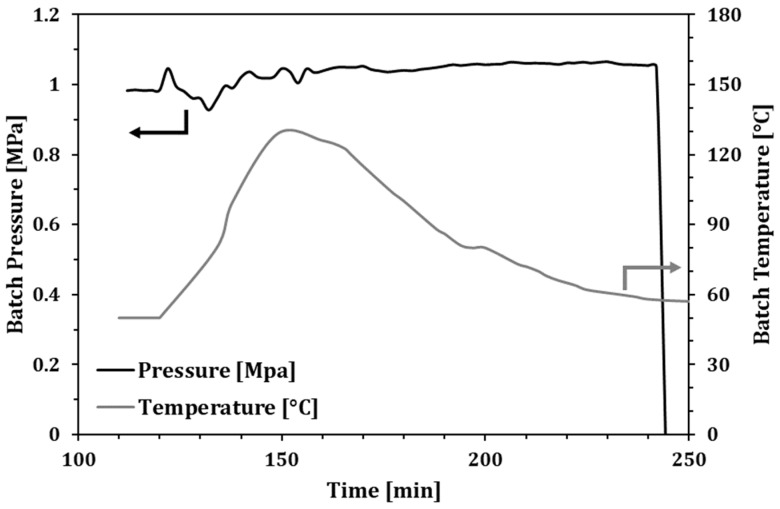
Batch pressure and temperature along Exp. 2.

As shown in Figure 2, an initial peak a quick decrease was recorded in both temperature and gaseous reactants consumption: this is possibly due to the catalyst deactivation induced by the high temperature. 

**Figure 3:**
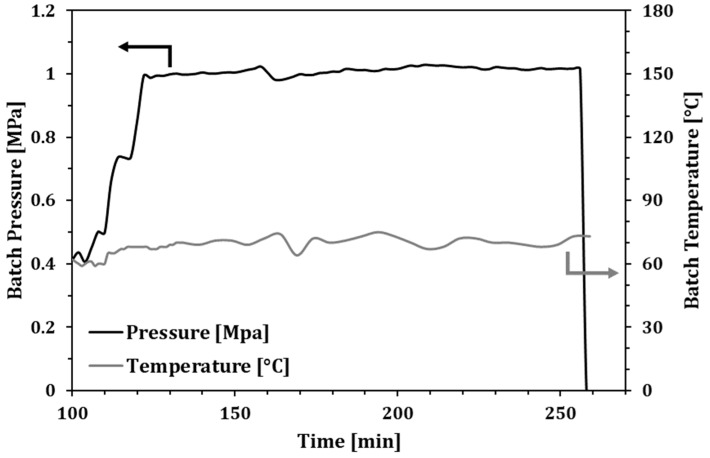
Batch pressure and temperature along Exp. 3.

With the aim of better controlling the process of exothermicity, Exp. 3 was set by using a minor amount of catalytic complex (6.6 mg) and was trigged with an operative pressure of 0.5 MPa that was increased in a stepwise manner up to 1 MPa in a few minutes. Such an approach avoids a sharp temperature increase, assuring a more uniform temperature trend throughout the test. Consequently, the smoothing of the temperature peaks allows for a more controlled reaction rate that ranged around 0.5 mmol s^−1^ L^−1^ in the whole investigated period, also resulting in a more stable catalyst activity, and with some ethylene consumption still recorded at the end of the test (Figure 3). It was also noticeable how system pressure raising (in the first 20 min of the test) involves a considerable increase in the reaction rate, from around 0.22 mmol s^−1^ L^−1^ up to more than 0.76 mmol s^−1^ L^−1^.

In terms of process yield, the better control of the process achieved by Exp. 3 procedure allowed for an overall better performance, since around 22 g of ethylene were converted by the catalytic system, while the Exp. 2 procedure reduced the ethylene consumption to around 14 g, probably due to an earlier catalyst deactivation.

At the end of experimental tests, the reaction mixture was analysed after some physical treatments. A waxy semisolid was recovered by filtration, while the liquid phase was shaken with water and hexane and then the organic phase dried over magnesium sulphate. Finally, raw oil was obtained by removal under a reduced pressure of the volatile components.

**Figure 4:**
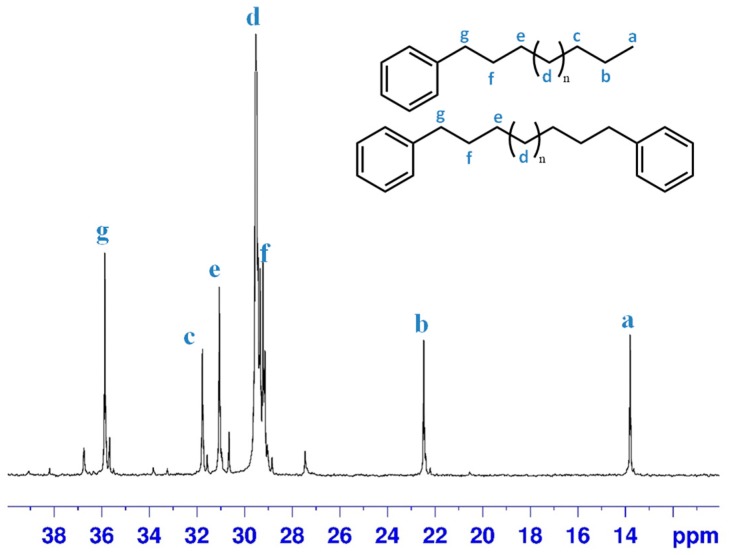
^13^C-NMR spectra of wax produced in Exp. 3 (aliphatic region).

The semisolid material was analysed by a ^13^C-NMR analysis; the aliphatic region of the spectrum is reported in Figure 4. By assuming the absence of n-alkanes, it is possible to estimate around 3.2 the ratio of LAB or DPA. It is worth noting how in Exp. 2 and Exp. 3, in contrast to Exp.1, no traces of polyethylene were detected, probably as a further effect of the better control of the composition of the gaseous phase. 

**Figure 5:**
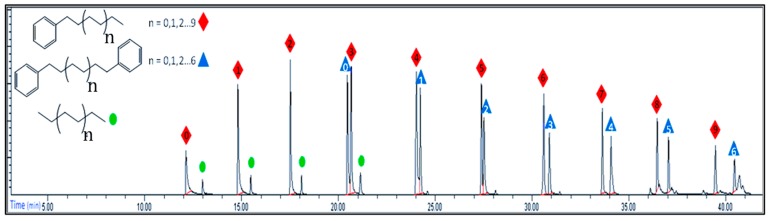
GC of raw oil (Exp. 3).

The GC traces (Figure 5) of the viscous oil show two main classes of compounds, the LAB and the α–ω DPA, in addition to a very small amount of ethylene oligomers. 

## 3. Discussion

Based on previous studies [6,7,9], it is possible to argue that LABs probably derive from a primary styrene insertion into the Zr-H bond, followed by some ethylene insertion, and culminated by the cutting of a metal-carbon bond by the molecular hydrogen; this last step in α–ω DPA formation is replaced by secondary styrene insertion, which presumably generated a catalytic site in a “dormant state” undergoing molecular hydrogen cutting.

The analysis of the produced mixture from the experimental tests is summarized in Table 1. The main evidence of the optimization of the process is the dramatic increase of the turnover on the catalytic sites that results one order of magnitude higher than the reference test (Exp. 1). In addition, the better management of the gas phase also produces a higher selectivity with a reduced production of α–ω DPA, all the while avoiding any production of PE. The analysis of the reaction rates (Figure 2 and Figure 3) also highlights how Exp. 2 suffered from a poor temperature control, which was possibly responsible for earlier catalyst deactivation and in turn also affecting the reaction turnover. On the contrary, in Exp. 3, in which lesser amounts of catalyst were used with respect to Exp. 2, a larger amount of oil was produced. In addition, by analysing the reaction rate trend (Figure 3), it is possible to argue that the catalyst did not suffer for relevant deactivation, still showing a noticeable catalytic activity at the end of the test. 

Finally, regarding per mmol of catalyst, it is possible to evaluate a turnover of 194 g per hour of the LABs with an aliphatic chain length, ranging from 4 to 22 carbon atoms. By considering that an average number of six molecules of ethylene inserts into the Zr-CH_2_CH_2_C_6_H_5_ bond before the cutting by hydrogen, it is possible to evaluate the relative reactivity of hydrogen and ethylene, in the assumption of a first order kinetics for both reactants. Thus, by combining this number with the ratio of the concentration of hydrogen (23 mM) [10] and ethylene (0.37 M) a r(H_2_,C_2_H_4_), a value = 2.7 can be obtained.

In conclusion, the use of a purposely designed reactor in conjunction with the accurate control of gas supply results in a good conversion of ethylene, styrene, and hydrogen to linear 1-alkylbenzenes, opening up to a possible upgrading of the synthetic process undertaken. In the approach described, the presence of by-products such as ethylene oligomers and α–ω diphenyl alkanes is dramatically reduced, with there also being a significant increase of reaction turnover (about 1 order of magnitude). The results reported confirm that a homogeneous poly-insertion catalysis can be a helpful tool for the synthesis of LABs under particularly mild operating conditions and from reagents that are generally available and inexpensive. 

## 4. Materials and Methods

### 4.1. General Procedures and Materials

All the manipulations of the air sensitive reagents were carried out under a dry nitrogen atmosphere using glove-box and Schlenk techniques. Toluene was kept refluxing 48 h over metallic sodium, and then distilled under a nitrogen atmosphere. Styrene was treated with calcium hydride, and then distilled under reduced pressure before use. Ethylene polymerization grade and hydrogen were used without further purification. Ethylenebis(tetrahydroindenyl) zirconium dichloride [11] was purchased from Sigma Aldrich (St. Louis, MO, USA). The modified methylaluminoxane (MMAO-12) was used as a dry powder obtained by distilling off under reduced pressure the solvent and the free Al(CH_3_)_3_ from the commercial modified methylaluminoxane (7% toluene solution, Sigma Aldrich).

### 4.2. GC-MS Analysis 

A dose of 1.0 μL of a 10% solution of the samples in CHCl_3_ was injected (split 1:10) into an Agilent 7890a GC (Santa Clara, CA, USA) (DB-17ms GC Column, 30 m, 0.25 mm, 0.25 µm, 7 inch cage). The GC instruments run with helium as a carrier gas at a constant flow rate of 1 mL/min. The injector temperature was set at 270 °C. The column temperature was initially kept at 40 °C for 2 min, and then increased from 40 to 170 °C at a rate of 10 °C/min. The column temperature was then maintained at 170 °C for 20 min, and was then increased from 170 to 280 °C at a rate of 10 °C/min. The detector Agilent mod. 5975C acquired the mass.

### 4.3. NMR Spectra

^13^C-NMR spectra in solution were recorded on a Bruker Avance 300-MHz spectrometer (Rheinstetten, Germany) (75.48 MHz for ^13^C) at 373 K with D1 = 2 s. The samples were prepared by introducing 25 mg of the wax in 0.5 mL of 1,1,2,2-tetrachloro-1,2-dideuterioethane (C_2_D_2_Cl_4_) into a tube (5 mm outer diameter). The chemical shifts refer to the central peak of C_2_D_2_Cl_4_ used as internal reference at ∂ = 74.26 ppm. 

### 4.4. Reactions under Pressure

The reactions were carried out in an autoclave, equipped with a bursting disk, magnetic stirrer, heating mantle, and pressure gauge. The reaction system consists of a cylindrical batch reactor realized in stainless steel AISI 316L, with a nominal diameter of 4′′ SCH5 (corresponding to an external diameter of 114.3 mm and a wall thickness of 2.11 mm), with a height of 104 mm, which assures a useful volume of around 1 L. The particular shape of the reaction system aims to maximize the gas–liquid exchange surface, and in turn the diffusion of gaseous reactants in the liquid phase. The reactor is able to sustain an operating pressure of 2.6 MPa and an operating temperature of 456 °C. The system was designed to allow the continuous monitoring of pressure, temperature, and gas phase composition. 

The reaction was charged with 200 mL of a mixture 7/13 *v*/*v* of styrene containing the co-catalyst (modified methylalumoxane) dissolved in it. After a preliminary procedure aimed at purging the reactor from any gas, the vessel was pressurized up to 1 MPa with an ethylene or hydrogen mixture. Once the system reached the desired pressure and temperature, the reaction was started by inserting the catalyst in the liquid phase through a pneumatic system. The catalytic complex was ETBHIZrCl_2_, and the amount loaded in the reaction system changed along the different tests, by holding the co-catalyst/catalyst ratio ([Al]/[Zr] = 400).

The hosted reaction converted gaseous reactants to liquid products, theoretically causing a system depressurization; moreover, reaction stoichiometry could vary during the tests depending on the system selectivity. Gaseous reactants, through a couple of mass flow controllers (Bronkhorst), were constantly fed into the system to keep constant the system pressure and gaseous phase composition. A gas analysis was carried out by a Quadrupole Dycor Dymaxion Mass Spectrometer provided by Ametek. The system was tuned to monitor mass 2 and mass 28 for the evaluation of hydrogen and ethylene volumetric fractions; the interference of ethylene on mass 2 was also considered. This configuration was able to either control the operating pressure of the reaction volume, or hold the desired gas composition, thus avoiding any disproportion between hydrogen and ethylene.

At the end, the gaseous mixture was vented off, and the liquid phase was poured into methanol (200 mL) acidified with about 4 mL of 37% hydrochloric acid. Through a filtration, a waxy semisolid was recovered, while the liquid phase diluted with hexane was shaken with water and then dried over magnesium sulphate. The low-boiling components was distilled off under reduced pressure to leave the viscous oil. 

## Figures and Tables

**Table 1:** Outlook on the performances of the catalytic complex EBTHIZrCl_2_ activated by MMAO.

Experiment ^a^	Turnover ^b^ (g/mmol × h)	Reaction Time (h)	Oil (g)	% LAB^c^	% α–ω DPA ^c^	Wax (g)	PE (g)
Exp. 1	30	1	0.48	55	37	-	0.15
Exp. 2	120	2.5	7.50	69	27	3.50	-
Exp. 3	285	2.5	11.00	68	30	6.50	-

^a^ The reaction parameters are described in the experimental section. ^b^ The turnover is reported as grams of raw oil per mmol of the ansa-metallocene complex per hour. ^c^ The complement to 100 is due mainly to ethylene oligomers and, for the second experiment, to trimers of styrene.

## Supplementary Materials

The supplementary materials are available online.

## References

[1] Kocal J., Vora B., Imai T. (2001). Production of linear alkylbenzenes. Appl. Catal. A.

[2] Perego C., Ingallina P. (2002). Recent advances in the industrial alkylation of aromatics: New catalysts and new processes. Catal. Today.

[3] Aslam W., Siddiqui M.A.B., Jermy B.R., Aitani A., Cejka J., Al-Khattaf S. (2014). Selective synthesis of linear alkylbenzene by alkylation of benzene with 1-dodecene over desilicated zeolites. Catal. Today.

[4] Han M., Cui Z., Xu C., Chen W., Jin Y. (2003). Synthesis of linear alkylbenzene catalyzed by Hβ-zeolite. Appl. Catal. A Gen..

[5] Zuo W., Wong H.-W. (2017). Green synthesis of linear alkylbenzenes via Diels-Alder cycloaddition between furan and linear alkenes over niobic acid catalyst. Green Chem. Lett. Rev..

[6] Galdi N., Lamparelli D.H., Oliva L. (2016). One pot synthesis of linear1-alkylbenzenes from styrene, ethylene and hydrogen. J. Mol. Catal. A.

[7] Galdi N., Buonerba A., Oliva L. (2016). Olefin-styrene copolymers. Polymers.

[8] Wu J., Pan Q., Rempe G.L. (2005). Solubility of ethylene in toluene and toluene/styrene-butadiene rubber solutions. J. Appl. Polym. Sci..

[9] Galdi N., della Monica C., Spinella A., Oliva L. (2006). Enantioselective C-C bond formation in styrene dimerization with chiral ansa zirconocene-based catalyst. J. Mol. Catal..

[10] Yin J.-Z., Tan C.-S. (2006). Solubility of hydrogen in toluene for the ternary system H_2_ + CO_2_ + toluene from 305 to 343 K and 1.2 to 10.5 MPa. Fluid Phase Equil..

[11] Wild F.R.W.P., Wasiucionek M., Huttner G., Brintzinger H.H. (1985). Ansa-metallocene derivatives: VII. Synthesis and crystal structure of a chiral ansa-zirconocene derivative with ethylene-bridged tetrahydroindenyl ligands. J. Organomet. Chem..

