# Supplementary material for: High Conversion of Styrene, Ethylene, and Hydrogen to Linear Monoalkylbenzenes
Source: Molecules. 2018 May 25;23(6):1260. doi: 10.3390/molecules23061260 (PMC6100200; doi:10.3390/molecules23061260)

Supplementary Materials

Figure S1. DSC thermogram of polymer material recovered as by-product in EXP1.

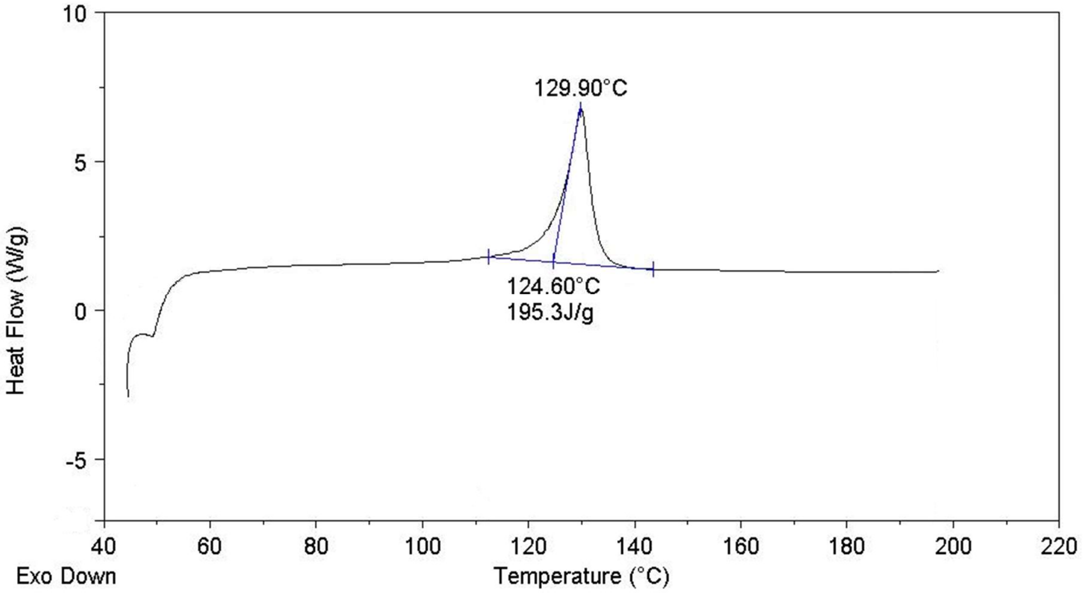

Figure S2. X-ray diffraction patterns for polymer material recovered as by-product in EXP1.

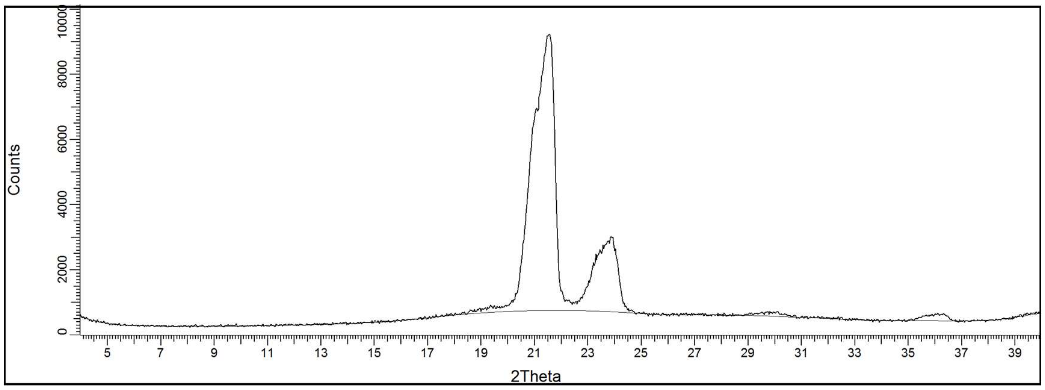

Supplement: Supplementary file 1 [file molecules-23-01260-s001.pdf]
